# Novel use of deep neural networks on photographic identification of epaulette sharks (*Hemiscyllium ocellatum*) across life stages

**DOI:** 10.1111/jfb.15887

**Published:** 2024-08-10

**Authors:** Martina Lonati, Mohammad Jahanbakht, Danielle Atkins, Stacy L. Bierwagen, Andrew Chin, Adam Barnett, Jodie L. Rummer

**Affiliations:** ^1^ College of Science and Engineering James Cook University Douglas Australia; ^2^ Marine Data Technology Hub James Cook University Townsville Australia; ^3^ AIMS@JCU Townsville Australia; ^4^ Centre for Tropical Water and Aquatic Ecosystem Research (TropWATER) James Cook University Douglas Australia; ^5^ AIMS@JCU, Division of Research and Innovation James Cook University Townsville Australia; ^6^ Australian Institute of Marine Science Cape Cleveland Australia; ^7^ Biopixel Oceans Foundation Cairns Australia

**Keywords:** artificial intelligence, deep learning, elasmobranch, *Hemiscyllium ocellatum*, machine learning, non‐invasive, photo ID

## Abstract

Photographic identification (photo ID) is an established method that is used to count animals and track individuals' movements. This method performs well with some species of elasmobranchs (i.e., sharks, skates, and rays) where individuals have distinctive skin patterns. However, the unique skin patterns used for ID must be stable through time to allow re‐identification of individuals in future sampling events. More recently, artificial intelligence (AI) models have substantially decreased the labor‐intensive process of matching photos in extensive photo ID libraries and increased the reliability of photo ID. Here, photo ID and AI are used for the first time to identify epaulette sharks (*Hemiscyllium ocellatum*) at different life stages for approximately 2 years. An AI model was developed to assess and compare the reliability of human‐classified ID patterns in juvenile and neonate sharks. The model also tested the persistence of unique patterns in adult sharks. Results indicate that immature life stages are unreliable for pattern identification, using both human and AI approaches, due to the plasticity of these subadult growth forms. Mature sharks maintain their patterns through time and can be identified by AI models with approximately 86% accuracy. The approach outlined in this study has the potential of validating the stability of ID patterns through time; however, testing on wild populations and long‐term datasets is needed. This study's novel deep neural network development strategy offers a streamlined and accessible framework for generating a reliable model from a small data set, without requiring high‐performance computing. Since many photo ID studies commence with limited datasets and resources, this AI model presents practical solutions to such constraints. Overall, this approach has the potential to address challenges associated with long‐term photo ID data sets and the application of AI for shark identification.

## 1 INTRODUCTION

In ecological research, identifying and counting individuals within a species is often the first step to understanding population dynamics. Long‐term monitoring of aquatic species has traditionally used capture‐mark‐recapture (CMR) methods, which involve tagging or “marking” of individual organisms to differentiate them within their population, facilitating subsequent re‐captures over time and the reconstruction of re‐encounter histories (Jolly, 1965; Musick & Bonfil, 2005; Pine et al., 2003). Data derived from CMR studies are then used to estimate population sizes and collect information on life‐history parameters, animal movement, and habitat use (Cameron et al., 2019; McCoy et al., 2018; Peterson & Grubbs, 2023). However, CMR methods require the physical capture and handling of individuals, posing logistical challenges, potential danger for the animal and the researchers, and possibly disrupting natural behaviors (Bouyoucos et al., 2020; Pauli et al., 2010). Photographic identification (herein, photo ID) has emerged as a non‐invasive alternative, gaining traction alongside technological advancements in media data acquisition and processing (Miele et al., 2021; Pierce et al., 2019). Photo ID originated in the 1970s (Myrberg & Gruber, 1974) and applies image‐based biomonitoring (i.e., photographs). This method leverages unique and temporally stable biometric features similar to fingerprints, which are unique to an individual, thus helping identification (Jenrette et al., 2022).

Tracking the same individuals through time can be challenging in marine environments, particularly when targeting cryptic species that naturally occur in low abundance, spend most of their time at inaccessible depths, or constantly move across large distances (Harty et al., 2022; Jackson et al., 2006; Ramsey et al., 2019). The advent of underwater video technologies has assisted the increasing popularity and growing adoption of photo ID as a method for monitoring marine animals (Anderson & Goldman, 1996; Arzoumanian et al., 2005; Corcoran & Gruber, 1999; Hammond et al., 1990). This is particularly relevant for animals that are too large to be captured or for protected species where direct manipulation may be challenging. Among these species, there are several examples of elasmobranchs (i.e., sharks, skates, and rays) that have been studied using photo ID (Marshall & Pierce, 2012). Photo ID is arguably the standard method for monitoring white sharks (*Carcharodon carcharias*; Becerril‐García et al., 2020; Micarelli et al., 2021; Schilds et al., 2019), whale sharks (*Rhincodon typus*; Araujo et al., 2019; Arzoumanian et al., 2005), gray nurse sharks (*Carcharias taurus*; Bansemer & Bennett, 2008), Indo‐Pacific leopard sharks (*Stegostoma tigrinum*; Dudgeon et al., 2008), and manta rays (*Mobula alfredi and M. birostris*; Harty et al., 2022; Town et al., 2013). However, from the ~1200 species of elasmobranchs (IUCN SSC Shark Specialist Group, 2023), there are only a few examples of photo ID being used for other species, for example blacktip reef sharks (*Carcharhinus melanopterus*; Mukharror et al., 2019), basking sharks (*Cetorhinus maximus*; Gore et al., 2016; Southwood, 2008), great hammerhead sharks (*Sphyrna mokarran*; Guttridge et al., 2017), nurse sharks (*Ginglymostoma cirratum*; Castro & Rosa, 2005), white spotted eagle rays (*Aetobatus narinari*; Cerutti et al., 2018), bull rays (*Aetomylaeus bovinus*; Moreno et al., 2021), and several species of skates (Benjamins et al., 2018). Generally, photo ID has gained acceptance and traction as a reliable method for studying elasmobranch population dynamics and ecology (Pierce et al., 2019). Yet the low number of species studied implies that not all species are suitable for photo ID (Marshall & Pierce, 2012).

Certain conditions and assumptions need to be satisfied to achieve reliable and accurate results in photo ID studies. One of the main conditions for photo ID is that morphological features used for identification must be both discernible and stable through time, and several species of elasmobranchs exhibit natural patterns conducive to ID (Armstrong et al., 2020; Arzoumanian et al., 2005; Harty et al., 2022). However, only a few studies have attempted to validate photo ID through multi‐modal methodologies or by convergent evidence from different markers, including sex and external tags (Bansemer & Bennett, 2008; Dudgeon et al., 2008; Gubili et al., 2009; Winton et al., 2023). For example, white sharks are identified by the trailing edge of their dorsal fin, which may seem quite similar among individuals, but on closer examination it carries substantial individuality (Andreotti et al., 2014). When photo ID is used as a CMR method for long‐term studies, the stability of patterns is often inferred via observational evidence, but this is rarely subjected to a systematic, longitudinal evaluation (Bègue et al., 2020; McCoy et al., 2018; Pratt Jr. et al., 2022; Winton et al., 2023). Additionally, in species that exhibit ontogenetic changes in their morphology, such as Indo‐Pacific leopard sharks (*Stegostoma fasciatum*) and tiger sharks (*Galeocerdo cuvier*; Fu et al., 2016), the presence and persistence of patterns during early life stages constitute a species‐specific variable that is not universally addressed, thereby constraining the applicability of photo ID to mature life stages for most species (Marshall & Pierce, 2012). Another essential consideration is the approachability of the species in their natural habitat. While contemporary underwater video technologies can access a broad range of environments and depths, optimal photograph quality is typically achieved at shallow depths and in clear waters (Deakos et al., 2011; Marshall et al., 2011; McCoy et al., 2018). Consistent or well‐defined seasonal aggregations of certain species in known locations facilitate longitudinal, photographic documentation, thereby enhancing the reliability of re‐sightings for population modeling (Changeux et al., 2020; Marshall & Pierce, 2012; Pratt Jr. et al., 2022). Some elasmobranch species, such as manta rays, whale sharks, and white sharks, meet the assumptions and conditions for successful photo ID, resulting in large‐scale, multilocation, long‐term projects that compile extensive datasets of photographs through time (Araujo et al., 2017; Armstrong et al., 2019; Norman et al., 2017). For example, the Wild Book for whale sharks (McCoy et al., 2018) and the Manta Matcher (Town et al., 2013) are examples of open‐access global datasets of photographs that can be collected by anyone, anywhere.

To expedite processing times and reduce the potential for human error, many photo ID projects have implemented automated or semi‐automated image analysis through artificial intelligence (AI; Carter et al., 2014; Miele et al., 2021; Weinstein, 2018). As an example, open access platforms such as WildMe (https://www.wildme.org/what-we-do.html) have developed AI‐powered computer vision technologies for some of the most iconic elasmobranch species to facilitate individual recognition, such as Sharkbook and Manta Matcher (Conservation X Labs, 2024). These platforms are under continuous maintenance to develop new solutions and improve the speed, automation, and accuracy of identification. In computer technologies, AI refers to a goal, machine learning is a technique, and deep learning is a tool, but these terms are used interchangeably in the literature. Machine learning refers to a type of algorithm that can autonomously identify patterns in data, even when data are nonlinear and complex, and can create predictive models (Christin et al., 2019). The application of machine learning in computer vision goes back to simple statistical modeling and shallow neural networks (Hu et al., 2012), but this technology has recently evolved into advanced, deep neural networks (DNN; Jahanbakht et al., 2022). Most photo ID projects on elasmobranchs have used shallow neural networks, heavily relying on researchers' expertise to perform feature extractions and labelling (Andreotti et al., 2018; Arzoumanian et al., 2005; Hughes & Burghardt, 2017; Town et al., 2013). The more advanced DNNs use a function approximator called a neural network that contains different modifiable parameters organized in layers of neurons. These layers can receive the data, process it (within the processing core), and give the result of the model (output layer). One of the most important advantages of DNNs in photo ID applications is the automated extraction of distinguishing features from an image. The feature extraction process takes place within the multiple layers of the processing core, where progressively more accurate outputs are given through a self‐improving learning process (LeCun et al., 2015). This is achieved by customizing one of the layers of the neural network for the image classification task. Specifically, the convolutional layer is used in convolutional neural networks (CNNs) and is often adopted for computer vision as it can extract specific patterns to classify images. CNNs have achieved the best performances in photo ID studies of turtles, great apes, giant pandas, and giraffes, but major limitations arise with small and unlabeled training datasets, temporal changes in morphology, and re‐identification (Christin et al., 2019; Miele et al., 2021). The automated re‐identification of previously known individuals is a necessary feature of AI applied to photo ID. Currently, most models rely on an extensive library of photographs for training, and as a new individual is photographed, the model needs to be completely retrained (Schneider et al., 2022). Similarity comparison networks are a novel approach to addressing the issues of re‐identifying individuals from small and unlabeled datasets (Miele et al., 2021), which is often the best available resource when studying wild populations of elasmobranchs.

The aim of this study was to develop a framework to incorporate novel AI approaches in photo ID projects for long‐term monitoring of elasmobranchs. In photo ID studies, the species of choice needs to be easily distinguishable by its individually unique patterns. In this study, this requirement was fulfilled by choosing a species of elasmobranch, the epaulette shark (*Hemiscyllium ocellatum*), as the focal species and developing a photo ID protocol that incorporates both standard photo ID processes and innovative AI applications. Importantly, epaulette sharks change patterns as they mature from their neonatal stage, allowing this study to assess the implications of unstable patterns during early ontogeny (Ferreira et al., 2020). Focusing on a captive population of epaulette sharks, the model could be trained with photographs of known individuals, from all life stages, and at different times throughout the study. The same model was used to test if patterns change over time and whether immature life stages can reliably be identified from their patterns. The trial‐and‐error process in this study led to the final photo ID protocol and AI model, which represents an ideal trade‐off between simplicity, flexibility, precision, and AI innovation.

## 2 MATERIALS AND METHODS

### 2.1 Ethics

The care and use of experimental animals complied with the animal welfare laws determined by the Australian Code for the Use of Animals for Scientific Purposes, and the guidelines and policies as approved by the James Cook University Animal Ethics Committee (protocol A2826). The animals that were photographed in captivity were collected under the appropriate Great Barrier Reef Marine Park Authority (GBRMPA #G19/43380.1) and Queensland Fisheries (#200891) permits.

### 2.2 Photographing sharks

**FIGURE 1:**
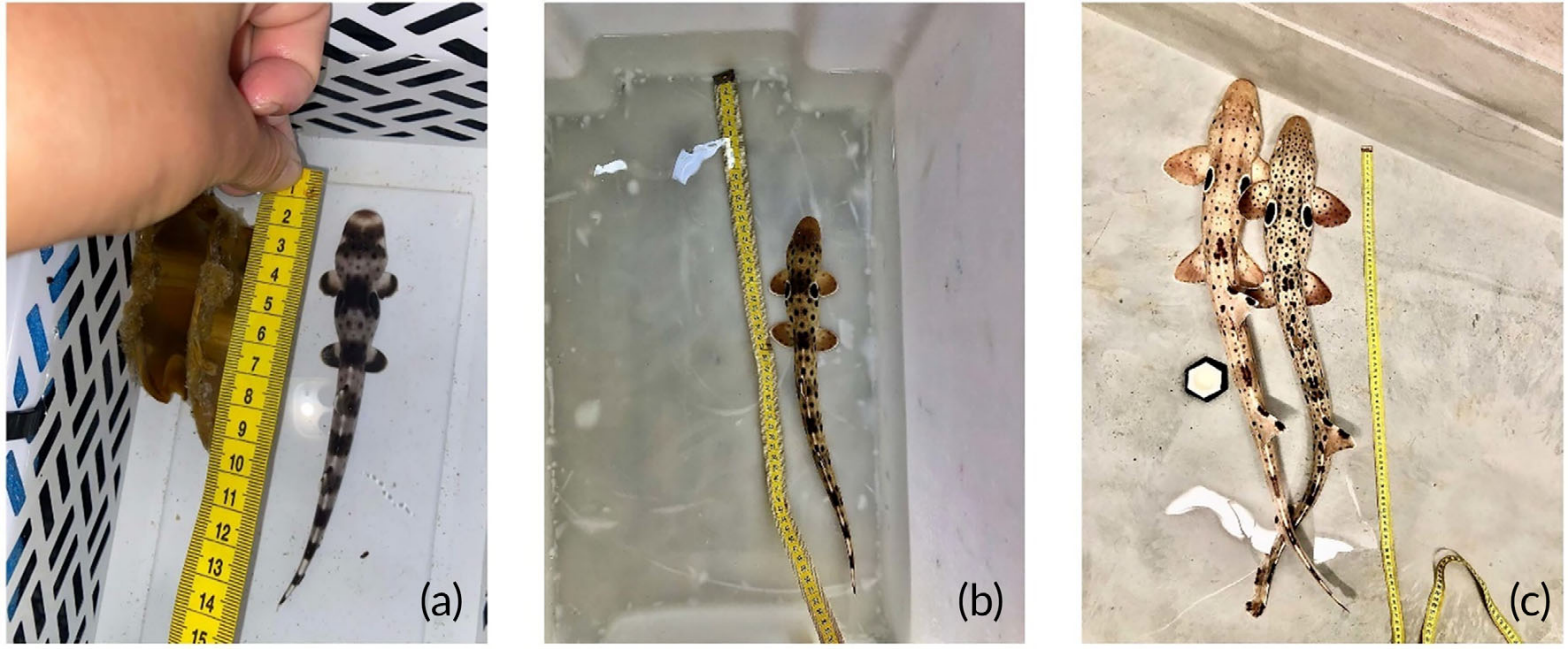
From the left, typical neonate (a), juvenile (b), and adult (c) epaulette sharks (*Hemiscyllium ocellatum*).

Epaulette sharks have a distinctive and individually unique pattern that can be used to distinguish adult individuals from each other. On the other hand, newborn and juvenile individuals can be harder to identify, as their pattern is constantly changing as they mature (Figure 1) (Payne, 2012). However, to date, there has been no scientific study that systematically observes and annotates how the patterns change with growth, and all available information has only been from anecdotal observations in captive environments. The changes observed in the patterns of young epaulette sharks provided an opportunity to test the stability of patterns for non‐mature individuals. In the current study, eight adults, four juveniles, and five neonates were photographed at the Marine and Aquaculture Research Facility Unit (MARFU) at James Cook University. The sharks photographed in this study were maintained in a controlled environment and separated in different tanks, allowing researchers to easily distinguish known individuals with their respective ID number. Additionally, the controlled environment provided a convenient setting for repeated photographic documentation of known individuals (Bansemer & Bennett, 2008). Also, the quality of photographs can be ensured, and unusable photographs can be re‐taken. This is not always feasible with wild populations due to varying conditions, including low visibility, surface and underwater photography, and the use of different camera types. To account for that, photographs for this study were taken with different angles, light availability, and all sorts of devices were used, from phones to professional cameras. This approach provided the AI model with a sufficient degree of variation in the examples used for training. In turn, this would give the model a certain degree of flexibility in what type of photographs can be usable for training. An additional difference between working with a captive population and a wild population is that captive populations are often closely monitored, sharks are known by ID, and records of labeled photographs are often available. These favorable conditions provide the AI model with a good base of information to start training. On the other hand, training a model for a wild population might be an ongoing and continuously updating process, as new individuals enter the population and need to be identified. To overcome the inconvenience of having to re‐train the model every time a new individual is added, this model implemented a similarity network approach similar to the one used in past studies (Schneider et al., 2022).

### 2.3 Dataset organization

**FIGURE 2:**
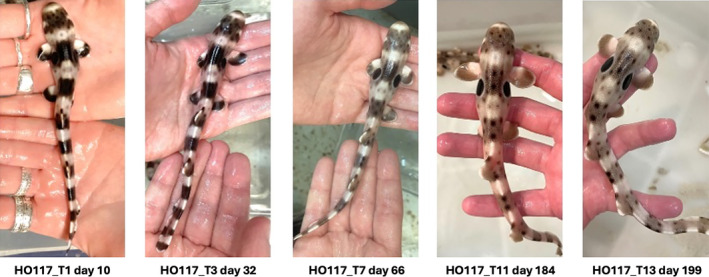
A typical neonate growth and change in pattern morphology in approximately 6 months.

Two series of photographs were taken: the baseline series and the time series. The baseline series comprised photographs of all sharks, and each shark was photographed multiple times on the same day. These photographs were classified by ID into the baseline dataset. The purpose of this dataset was to have multiple photographs of the sharks to train and test the AI model. Additionally, because all photographs were collected on the same day, there were no confounding effects of morphological changes that could have occurred through time. The time series of photographs were classified in the time dataset. The time dataset consisted of temporally consecutive photographs of the same sharks and was employed to test the model's ability to compare photographs of the same individual over time to assess if morphological changes have occurred in the ID patterns over time. Each photograph in the time dataset was labeled with the shark's ID and a time marker, such as T0, T1, T2, T3, (…), Tn. Time intervals between consecutive photographs of adult and juvenile sharks were not standardized (see Table S1). This approach is relevant for the model's real‐world applicability, given that re‐sightings of wild elasmobranchs are unlikely to occur at regular intervals due to environmental factors. However, the time elapsed between consecutive photographs of neonate sharks was deliberately controlled to be taken within one week. However, some photographs were not usable due to poor quality, and had to be discarded. Nevertheless, the time elapsed between two consecutive photographs was never longer than one month. For instance, significant pattern changes were expected for neonate epaulette sharks within the first year post‐hatch (Figure 2), and photographs were taken often and regularly to capture such changes.

**TABLE 1:** Comparing the accuracy, precision, and mean absolute error (MAE) performances of different deep neural network models on multiple combinations of the testing dataset.

Image data	Evaluation	Model	Accuracy	Precision	MAE
All life stages	Image‐based	EfficientNetB4‐Head	47.00%	8.10%	0.53
		EfficientNetB4‐PEC	41.70%	8.20%	0.583
		EfficientNetB4‐FDF	44.90%	5.60%	0.551
		EfficientNetB4‐FDB	56.20%	7.10%	0.438
		*XGBoost*‐all patches	90.40%	9.40%	0.096
All life stages	Shark‐based	EfficientNetB4‐Head	46.20%	14.40%	0.538
		EfficientNetB4‐PEC	39.20%	14.80%	0.608
		EfficientNetB4‐FDF	37.40%	10.60%	0.626
		EfficientNetB4‐FDB	53.80%	13.30%	0.462
		*XGBoost*‐all patches	90.10%	47.20%	0.099
Adult only	Image‐based	EfficientNetB4‐Head	71.30%	31.20%	0.287
		EfficientNetB4‐PEC	75.00%	28.80%	0.25
		EfficientNetB4‐FDF	23.30%	14.60%	0.767
		EfficientNetB4‐FDB	65.40%	18.40%	0.346
		*XGBoost*‐all patches	85.30%	45.50%	0.147
Adult only	Shark‐based	EfficientNetB4‐Head	75.00%	47.10%	0.25
		EfficientNetB4‐PEC	75.00%	46.20%	0.25
		EfficientNetB4‐FDF	22.20%	22.20%	0.778
		EfficientNetB4‐FDB	40.40%	11.10%	0.596
		*XGBoost* – all patches	86.10%	−100%	0.139

### 2.4 Photograph enhancement and masking

**FIGURE 3:**
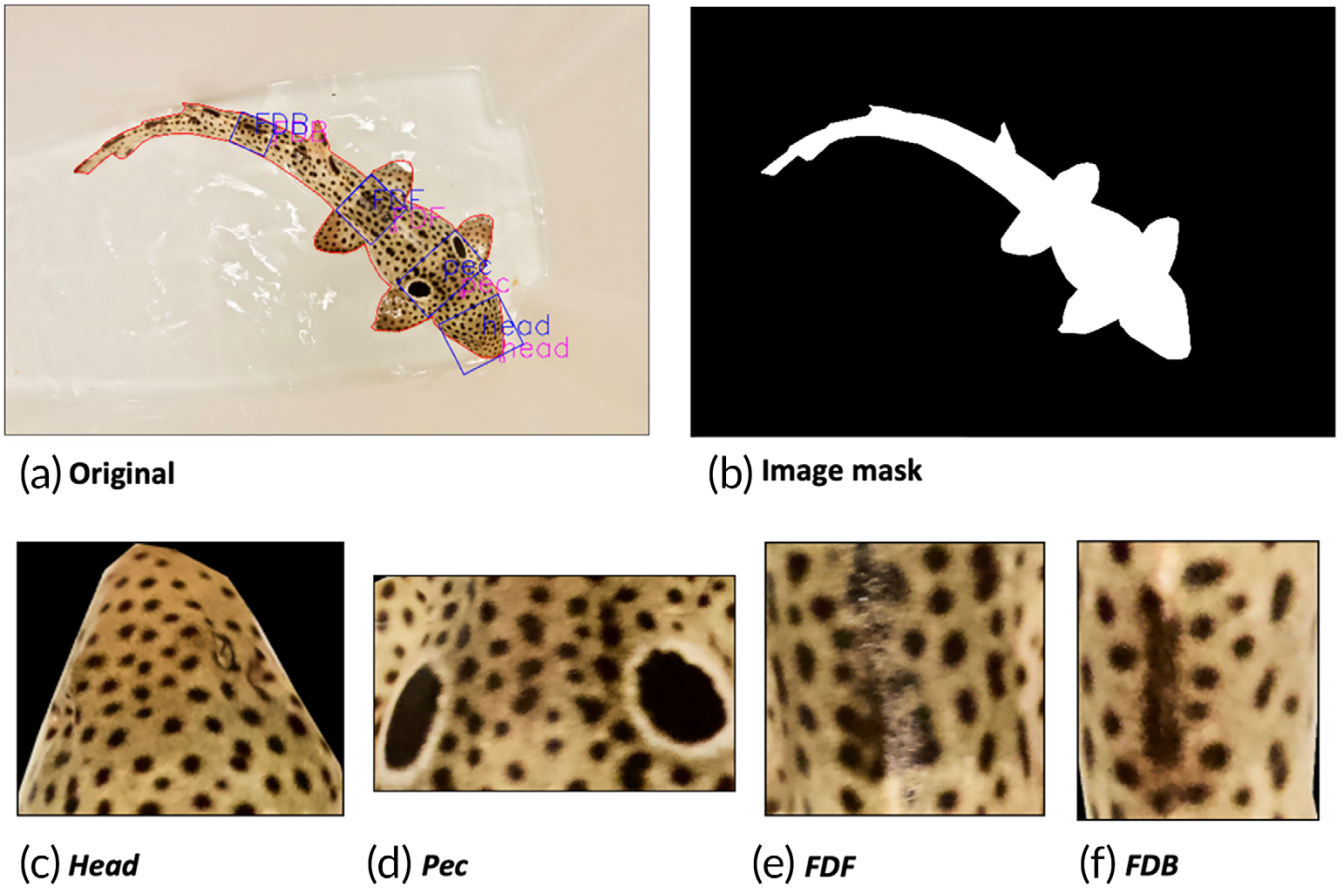
(a) Original image, (b) image mask, and (c, d, e, f) four subsampled image patches (head, pec, the dorsal area just ahead of the first dorsal fin [FDF], and the dorsal area just past the first dorsal fin [FDB]) of image HO_100_B0P2. These patches are sections of the sharks' body: (c) the head, (d) the area around the gills and pectoral fins, (e) the dorsal area in front of the first dorsal fin, and (f) the dorsal area behind the first dorsal fin.

Expanding on the dataset preparation, both the baseline and time datasets underwent a cleaning process. This involved removing duplicates (identical photographs) and blurry/low‐resolution photographs. Additionally, any photograph that did not display a dorsal orientation was excluded. Furthermore, each of the retained photographs was masked using the free program, Gimp – GNU Image Manipulation Program (version 21.0; GIMP Development Team, 2019). This masking technique is further illustrated in Figure 3a,b, where the visual focus is solely on the shark's silhouette, effectively eliminating any background distractions. Cropping the image to exclude the background facilitates the task of the AI model and decreases the amount of computational power needed. Generally, when processing images with low computational power (when high‐performance computing [HPC] is not available), it is best to pre‐edit the images used for training and testing. Cropping away the background and flagging identification features is more labor intensive but dramatically reduces the need for large computational power. This step can be avoided if HPC is available for image processing.

### 2.5 Skin boundary labelling

To accurately train deep learning models without overtraining, images were randomly grouped into batches of 16 images. The DNN's internal variables were gradually updated by these batches in an iterative process. Training an advanced DNN with batches of 16 high‐resolution images (i.e., 5472 × 3648 pixels) would typically require HPC, which is not readily available. To overcome this issue, four patches (i.e., body sections) from each shark image were subsampled (Figure 3c–f). These patches include the head (Figure 3c), pectoral/gills area (pec) (Figure 3d), the dorsal area just ahead of the first dorsal fin (FDF) (Figure 3, e), and the dorsal area just past the first dorsal fin (FDB) (Figure 3f).

By cropping and resizing the original images into four patches of 380 × 380 pixels, the need for HPC is eliminated. These body sections were chosen based on the experience of researchers and volunteers working in the laboratory where animals are routinely identified for measurements, feeding, and experiments. These four sections are thought to be the sections with most differences between individuals. The roLabelImg program (Cgvict, 2017) in Python (Van Rossum & Drake, 2009) was used to create “boxes” around these body sections (Figure 3a): The head region starts from the tip of the shark's nose, and down to approximately three‐quarters of the head length before the beginning of the pectoral region. The pec region starts from the top of the pectoral fin, behind the gills, to the base of the shark's black ocellus. The FDF region was designated as the heavily spotted region between the top of the pelvic fins to the base of the pelvic fins, found in front of the first dorsal fin. The FDB region was indicated by the densely spotted region between the base of the first dorsal fin and the front of the second dorsal fin. Four rotated rectangular boxes were drawn and tightened around the targeted skin patterns. Boxes were rotated to align with the direction of the vertebral line, then the front of each box was marked to indicate the direction toward the shark's head. This step was important to be able to align the boxes in the correct order of the respective body sections. This collection of boundaries was saved and uploaded with the respective labeled photographs and masks.

### 2.6 Image augmentation

**FIGURE 4:**
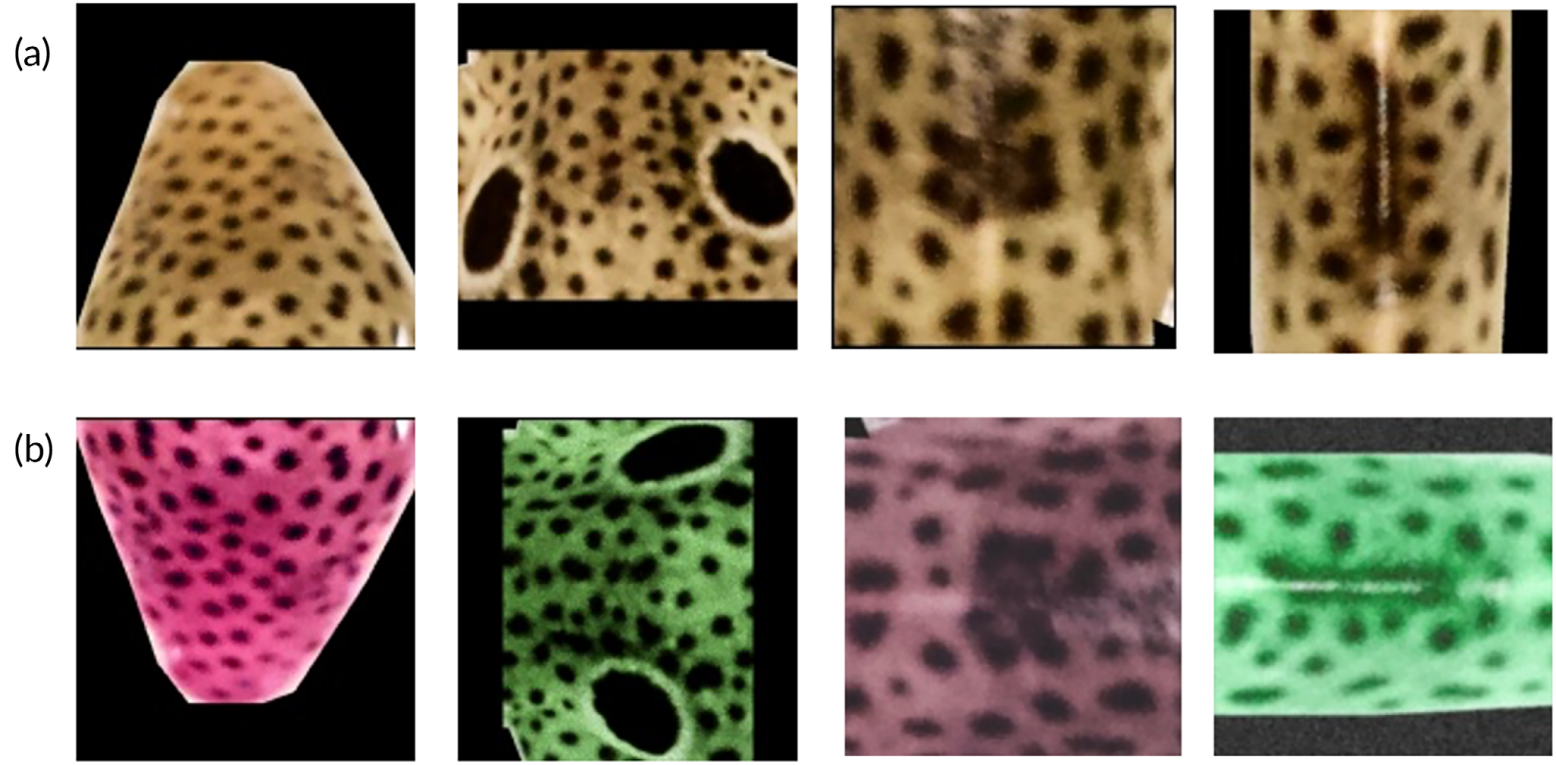
An example of random image augmentations for HO_100_B0P1. (a) Original head, pec, dorsal area just ahead of the first dorsal fin (FDF), and dorsal area just past the first dorsal fin (FDB) patches of the shark, and (b) head, pec, FDF, and FDB with random augmentations.

In the training phase of the DNN, photographs in the training dataset were randomly augmented to virtually increase their count and to avoid over‐ and undertraining. Each image was randomized with a series of flips (X‐flip and Y‐flip), rotations (90°, 180°, and 270°), Gaussian noise, Gaussian blur, gamma contrast, linear contrast, and other image manipulations. This image augmentation disturbs the RGB spectrum while keeping the sharks' skin patterns visually recognizable. This helps with training the model on shark skin patterns, instead of learning skin colors and color contrasts (Figure 4).

### 2.7 Training and testing

The AI model was initially trained and tested using the baseline dataset. When developing AI models, available photographs are subgrouped into training and testing datasets. This process is necessary to “teach” the model with similarities and differences for distinguishing individuals. From the baseline dataset, photographs of only six adults, three juveniles, and four neonates were used for training. It is important to note that not all the adults, juveniles, and neonates were shown during training. This process ensured that, when tested, the model looked for features within a new photograph, rather than remembering the previously seen the photographs themselves (Schneider et al., 2022). The remaining photographs not shown during training were grouped in the testing dataset, together with photographs of the remaining two adults, one juvenile, and one neonate. Furthermore, the time dataset was only used for the model testing. After the model had been trained on the basic task of distinguishing individual sharks, it was tested by presenting temporally consecutive photographs of the same shark from the time dataset. The purpose of this additional step was to test whether the morphology of the same shark changes through time enough so that the shark was not as distinguishable as it would be if no changes had occurred.

### 2.8 Model development

The DNN model used in this study was based on the EfficientNetB4, which is a CNN with optimum depth, width, and resolution scaling, introduced by Tan and Le (2019). Based on the benchmarking available in the Keras website (Chollet & others, 2015), EfficientNet and EfficientNetV2 are the most accurate models that offer small to extra‐large architectures with 29 to 479 MB size ranges. Among them, EfficientNetB4 was chosen as its 75 MB architecture fits well into an Nvidia GeForce RTX 2080 GPU processor. To train the proposed model with the limited baseline dataset (eight adults, four juveniles, and five neonates), a transfer learning strategy was employed. The EfficientNetB4 model was pre‐trained with 14,197,122 existing images of 1000 annotated objects in the ImageNet (Deng et al., 2021). The bottom layers of the pre‐trained model were then retained, and the top layer replaced with a customized new top. During the training, all 17,673,823 pre‐trained weights of the bottom layers were frozen, and only the 121,886 weights of the new top layer received training from the augmented images dataset.

**FIGURE 5:**
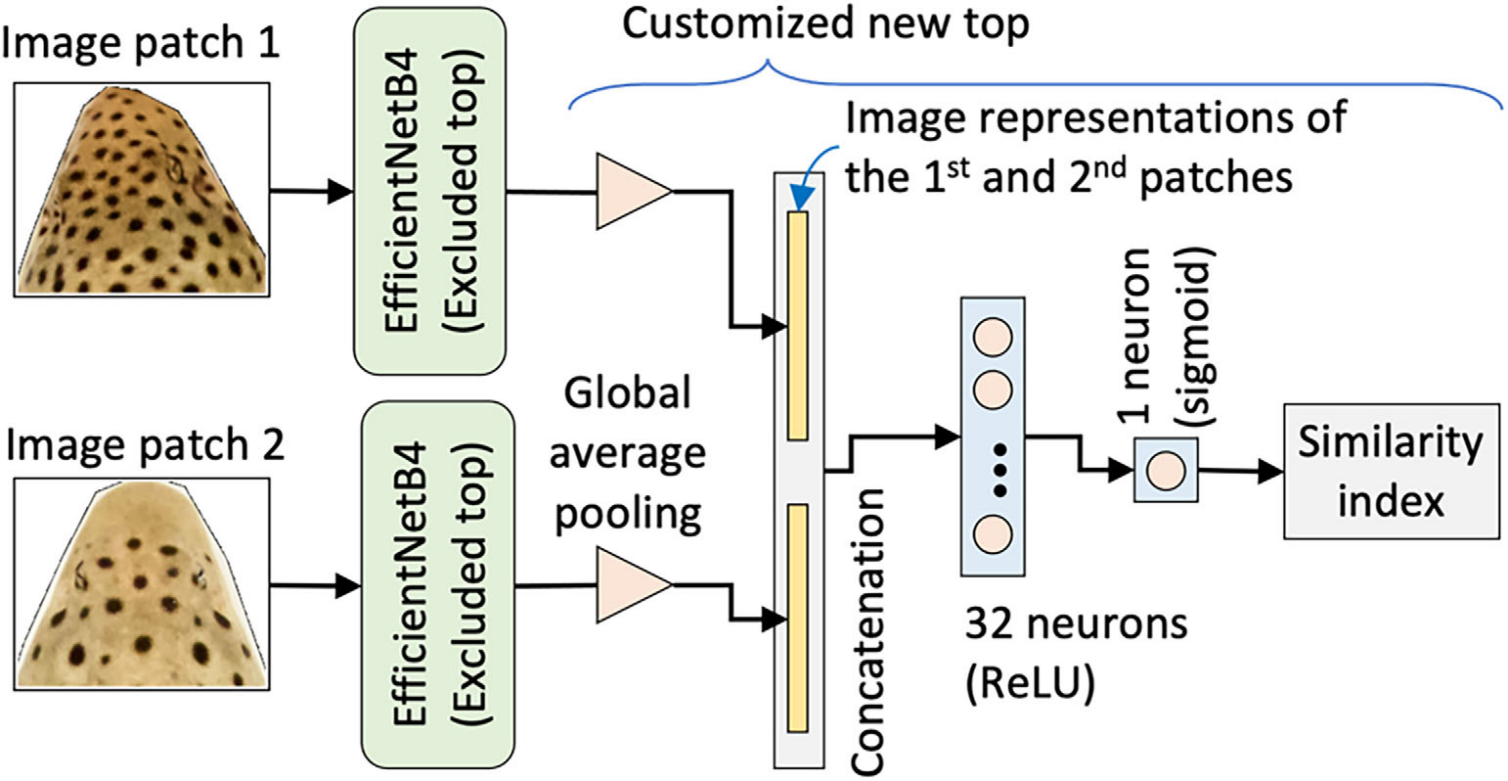
The proposed deep neural network, based on the EfficientNetB4 architecture, which receives two image patches, and returns their calculated similarity index.

The trained bottom layers were duplicated to form two identically frozen EfficienNetB4 models, each accepting an image patch at their inputs (Figure 5). The output of both models passed through an average pooling block to extract two image representations of the two input patches with a numerical vector that simply represents/encodes its relevant image. The two vectors were then concatenated into a single vector and passed through two subsequent dense layers. The first dense layer consisted of 32 fully connected neurons with rectified linear unit (ReLU) activation function. The second dense layer comprised one neuron with sigmoid activation to output a single probability‐like number between 0 and 1. This number is called the similarity index, and it shows the probability of the two input images being the same shark (1) or not (0). The larger the value, the higher the probability of two patches having similar skin patterns. The similarity index was adopted to identify single sharks by comparing the similarity indices returned during the analysis. Due to the limitation of only having 20 different sharks, the model was asked “Are these two sharks similar?” instead of “What is the ID of this shark?” This approach has been referred to as the similarity network approach and has been tested on several species, with photographs from open‐access datasets (Schneider et al., 2022).

### 2.9 Model assembling process

**FIGURE 6:**
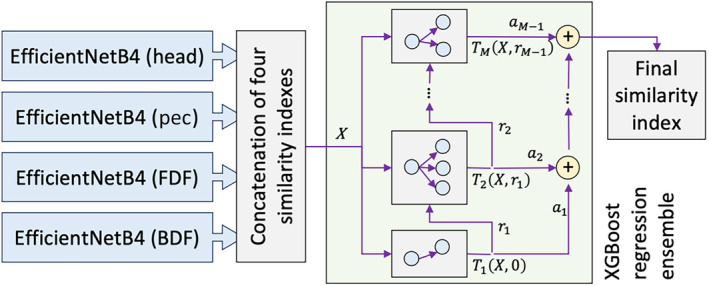
Merging the outputs of four independently trained deep neural network models of Figure 5 (on head, pec, dorsal area just ahead of the first dorsal fin (FDF), and the back dorsal fin (BDF), into a single *XGBoost* regression ensemble.

Using the four different patch types in the image dataset (i.e., head, pec, FDF, and FDB), four independent AI models were trained. With a further innovative step, the four AI models were ensembled (e.g., a combination of multiple algorithms) into a single decision‐making model. The chosen model was *XGBoost ensemble*, which has recently attracted more attention as a model assembling method (Jahanbakht et al., 2023). The four similarity indexes of the AI models were concatenated into X, which was then inputted into an *XGBoost* model and represented as a collection of M decision trees (Figure 6). The optimum value is automatically detected by the algorithm during the data fitting process (model training). Each decision tree i receives similarity indexes and returns $T_{i}\left(X,r_{i-1}\right)$, where $r_{i-1}$ is the residual output from the previous tree. The overall output of the *XGBoost* regression ensemble was then calculated as follows (Jahanbakht et al., 2023).

$${\left.\text{final similarity index}=\sum_{i=1}^{M}a_{i}T_{i}\left(X,r_{i-1}\right)\right|}_{r_{0}=0}$$

To infer an *XGBoost* model, images of two sharks were needed (either the same or different individuals). The head, pec, FDF, and FDB patches were extracted for each image, and the pairs fed into their relevant EfficientNetB4 model. This process generated the calculated final similarity index at the output of the *XGBoost* model. Any value greater than 0.5 (on a 0–1 scale) means that the input two images belong to the same shark; otherwise, they belong to different sharks. The process was named “image‐based evaluation”: one image of one shark is compared to another image of another shark. However, in this case, multiple images of the same shark are available, and a better‐performing process can be implemented. If $K_{1}$ images are confidently taken from shark 1 and $K_{2}$ images are taken from shark 2, then $K_{1}\times K_{2}$ is the number of different image pairs that can input the *XGBoost* model one by one. This resulted in $K_{1}\times K_{2}$ different final similarity indexes that were averaged to a number between 0 and 1. In contrast to the previous image‐based evaluation, this process is called “shark‐based evaluation.”

## 3 RESULTS

### 3.1 Dataset organization

The image‐based evaluation method was employed, where the similarity score is the result of the comparison between one image of one shark with only one other image of a different shark. The accuracy for the EfficentNetB4 – head/pec/FDF/FDB ranged between 47.0% and 56.2% accuracy, with low precision below 10% (Table 1: all life stages, image‐based). *XGBoost* showed a better performance (approximately 90% accuracy) as it combined all independent AI results into the final accurate decision on similarity or dissimilarity. Despite the accuracy increased by *XGBoost*, the precision was still lower than 10% (Table 1: all life stages, image‐based). This suggests that, although the approach is correct, there might be issues with the distinguishability of individuals, possibly arising from having only one image per shark with which to train.

To improve the precision of the models the shark‐based evaluation was applied to the first version of the models in the previous section. The shark‐based evaluation used the same datasets for training and testing but compared two groups of images (each group containing images of two different sharks), instead of one image per shark. Each group was previously classified to the correct ID in the training and testing datasets. All models increased precision by averaging over the multiple image pairs, with the *XGBoost* model as the most optimal with scores of 90% accuracy and 47.2% precision (Table 1: all life stages, shark‐based).

### 3.2 Testing the model: Can the model distinguish individuals?

**FIGURE 7:**
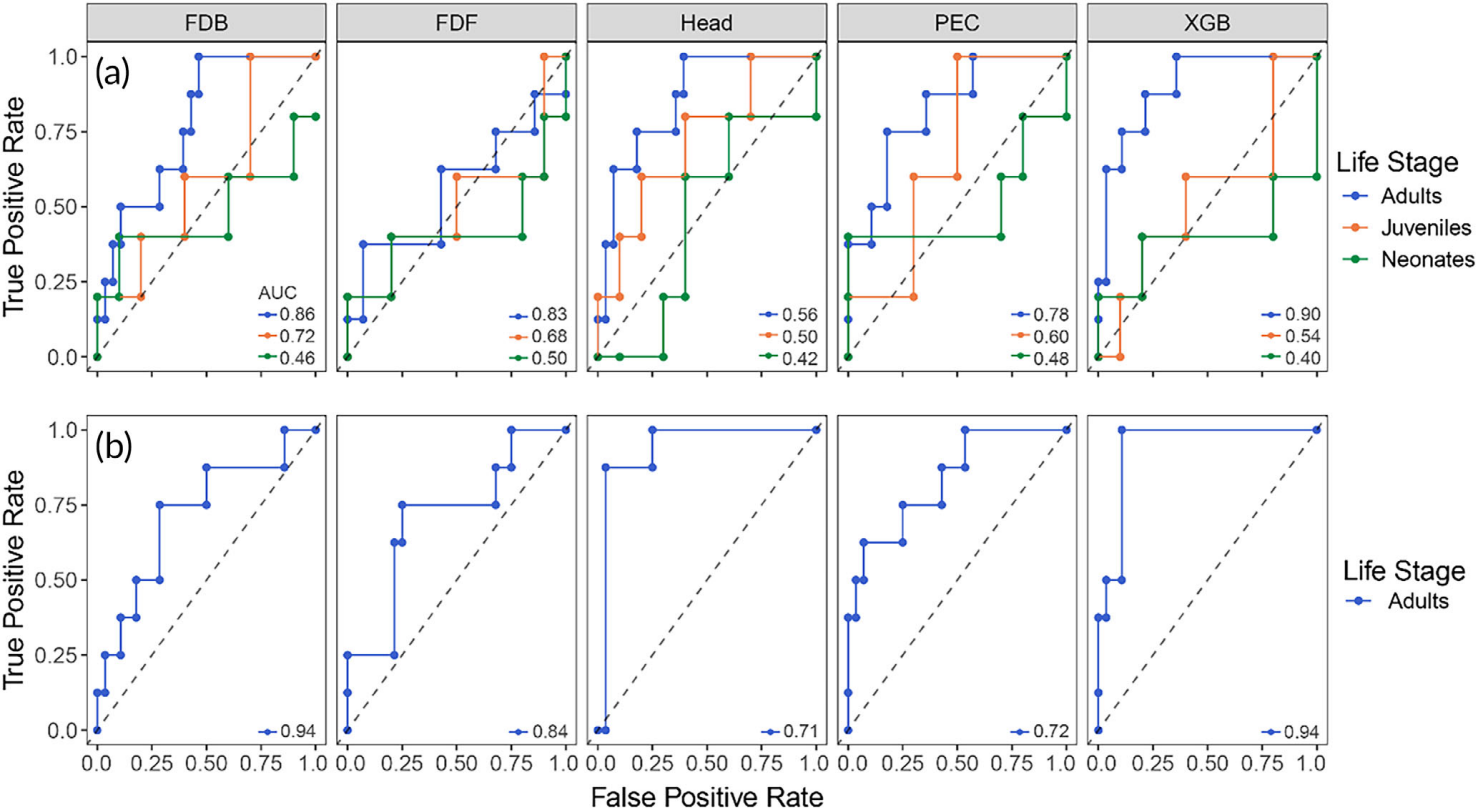
(a) Receiver operating characteristic curve for models trained on all life stages, and (b) on adults only. The area under the curve (AUC) represents the degree of separability between two classes (pairs of similar and dissimilar sharks in our case). The closer the value of AUC to 1, the better the model is at distinguishing two sharks from each other.

The receiver operating characteristic (ROC) curves show the performance of the models when distinguishing individuals (Figure 7). By adopting the similarity learning approach, the model was tested with the question “Are these two sharks similar or dissimilar?” rather than the question “Who is this shark?” This approach simplifies and streamlines the task by adopting an intuitive solution and asking to learn to distinguish individuals rather than learning their ID. Furthermore, this approach allows for any new animals to enter the population without having to re‐train the model to include a new ID (Schneider et al., 2022). In Figure 7, the area under the curve (AUC) values range from 0 to 1 and represent how well the model distinguishes two images.

When testing models on pairs of neonate sharks, the AUC values ranged from 0.50 for the model focusing on the pec area, to 0.40 for the *XGBoost* model (Figure 7a, neonates). The low AUC values indicate that there is no model available that is effective enough to distinguish neonate sharks from each other. The inability of the model to distinguish immature life stages is consistent with the experience of researchers who were tasked with the ID of neonate sharks. After repetitive ID sessions and several hours of sorting photographs, researchers succeeded in matching the ID to the right individual, mainly referring to the head area. Similarly, researchers were challenged with the ID of juvenile sharks (Figure 7a, juveniles and neonates). Models for juveniles showed some AUC improvement over neonates, ranging from 0.72 to 0.50, but were still considered to be poor determinants for differences among sharks. Overall, no model could reliably distinguish immature sharks from each other.

Alternatively, models trained on all life stages and tested on adult sharks showed considerable promise in the identification of differences between adult individuals. The head model and the *XGBoost* model retained the strongest AUC values, 0.86 and 0.90, respectively (Figure 7a, adults). The least accurate model was the FDF (0.56) model, followed by the FDB model (0.78) (Figure 7a, adults).

### 3.3 Training on adults‐only, image‐based, and shark‐based methods

Because the model is not able to differentiate immature individuals, the training of the first version of the models could have been affected by the presence of images that cannot be distinguished by the model. The presence of neonates and juveniles in the training datasets might have “confused” the model in the training phase. For this reason, neonates and juveniles were removed from the training dataset, and the second version of the models were trained on the adult sharks only. The precision increased from 9.4% of the previous image‐based model to 45.4% when the model was trained on adults only (Table 1: adult only, image‐based). However, the accuracy of the model decreased from 90.4% to 85.3% because of the smaller training dataset including only adult sharks. The best results overall were achieved when introducing the shark‐based evaluation to the model trained on adult sharks, reaching 86% accuracy and nearly 100% precision (Table 1: adult only, shark‐based). With this approach, the ROC curves for the head and pec models have AUC values of 0.93 and 0.83, respectively, while the *XGBoost* model seems to have the best results and an AUC value of 0.94 (Figure 7b). Intuitively, because the training for this last set of models was done on adults only, the model can only be tested on adult sharks.

### 3.4 Results of the temporal analysis

**FIGURE 8:**
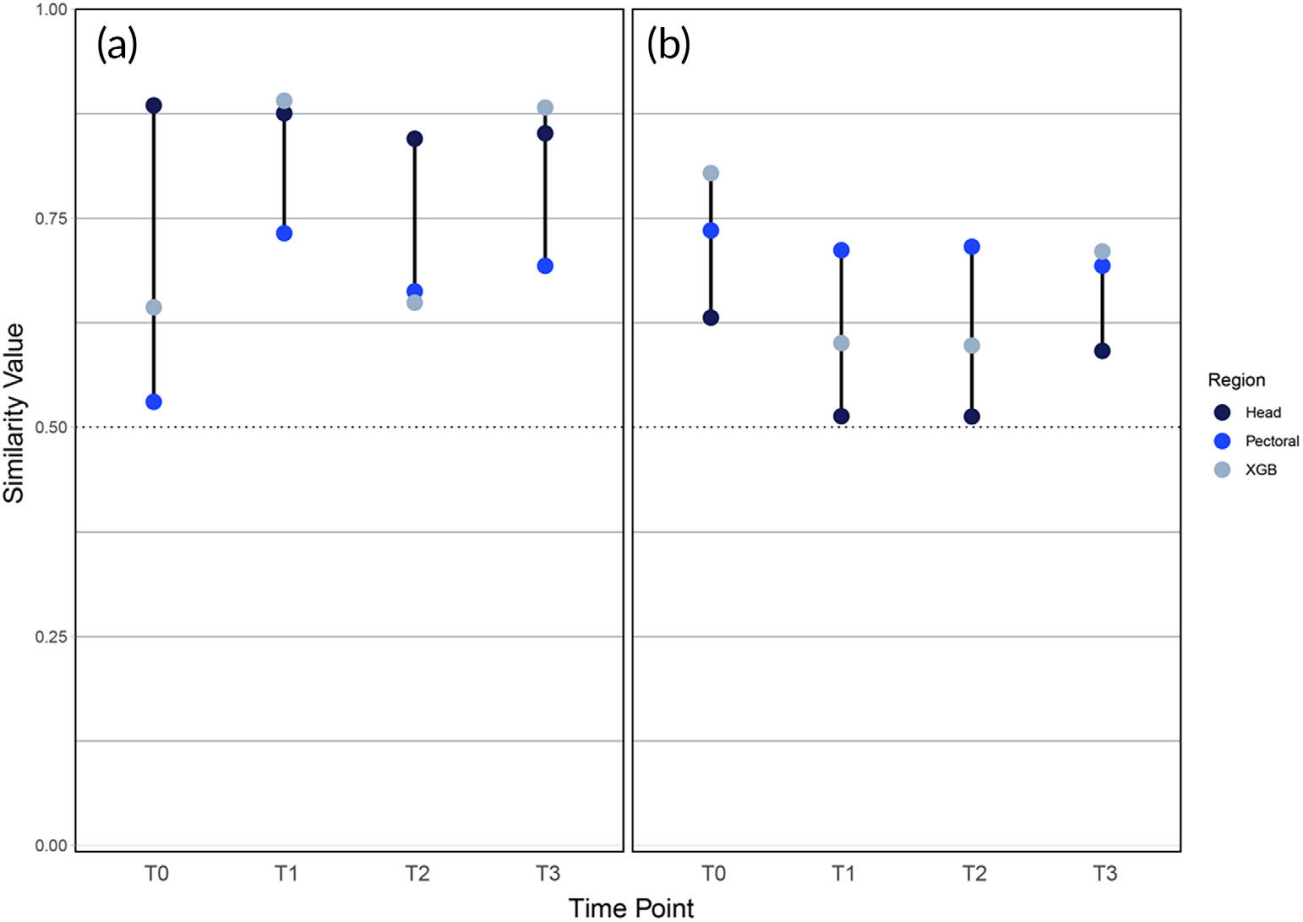
(a) Similarity indices of the image pairs of the same sharks through time (T0 to T3) for adult‐only, and (b) all life stages. A 50% similarity threshold is represented by a dotted line, where any AI output above this line means that image pairs correctly belong to the same shark.

Finally, with the same AI model design, the time dataset was used to assess substantial changes in the morphology by using images of the same adult shark through time. Each time step (T1, T2, T3, and T4) was measured against baseline images of the same shark, which were taken after all the photographs for the time dataset (Table S1). The analysis was limited to adult sharks, as the previous results have shown the AI model could not reliably distinguish juveniles and neonates from each other. The spot patterns of adult epaulette sharks are thought to be permanent, but the long‐term stability of patterns was never tested for adults, juveniles, and neonates. Results from the deep learning model confirm that the pattern morphology of adult sharks remained stable through the duration of the study (~21 months). The same model could be used with longer time intervals between photographs. Although a historical database of photographs could be insightful of longer‐term morphological changes, this study suggests that, once adult maturity has been reached, patterns can stabilize and remain the same through the lifetime of epaulette sharks. The effectiveness of the applied deep learning model is most evident with larger, historic database of photographs. In the case of long‐term database, this model could be applied across thousands of individuals and recognize small changes in morphology through time. Such changes would be represented by a small decrease in similarity score, indicating that the animal seems to be the same individual, but something has changed in its morphology. The best‐performing head, pec and *XGBoost* models were trained on adults only (Figure 8a) and on all sharks (Figure 8b). Each model returned similarity indexes above 0.5, but as expected, the models trained on adults only had better performance. Similarity values above 0.5 suggest that spot patterns do not change significantly. In terms of the performance of the models, when trained on all age groups (Figure 8b), pec is a better indicator of age's effect on skin pattern. When models are trained with adult sharks only (Figure 8a), the head area seems to be a better indicator of differences through time. *XGBoost* was determined to be a reasonable choice in both cases.

## 4 DISCUSSION

This study is the first to develop a photo ID protocol for epaulette sharks, contributing to the small list of species that can be effectively studied using photo ID. The protocol outlined in this study includes a series of innovative solutions in the field of applied AI to photo ID. First, the deep learning model was effectively trained on a small dataset of labeled images, the model was developed with a regular computer with standard computational power, and the model used the similarity network approach to ID individual sharks, overcoming the typical close‐population assumption of many photo ID models. Furthermore, the protocol was used to assess the reliability of patterns for immature life stages, and to detect changes in morphology through time. Because of the small number of images required, the proposed protocol provides a cost‐ and time‐effective tool to test the applicability of photo ID on new species of elasmobranchs and perhaps teleost fishes and cetaceans. The same method can be useful for species like manta rays and whale sharks that have been surveyed with photo ID for decades (Harty et al., 2022; McKinney et al., 2017). For instance, this new approach provides a tool to validate whether skin patterns change over long‐term studies by using the similarity network approach on large databases of photographs collected through time. The long‐term stability of ID patterns can also be confirmed by pairing photographs with genetic samples of individuals (Gubili et al., 2009), but genetic methods are not always logistically or financially possible. However, the reliable identification of individuals with morphology and the long‐term stability of such morphological traits are fundamental requirements for population studies using photo ID as a non‐invasive capture mark recapture method. The protocol proposed in this study represents an applicable set of solutions to photo ID and AI applications to animal identification and population studies.

### 4.1 A step‐by‐step approach to elasmobranch photo ID

**FIGURE 9:**
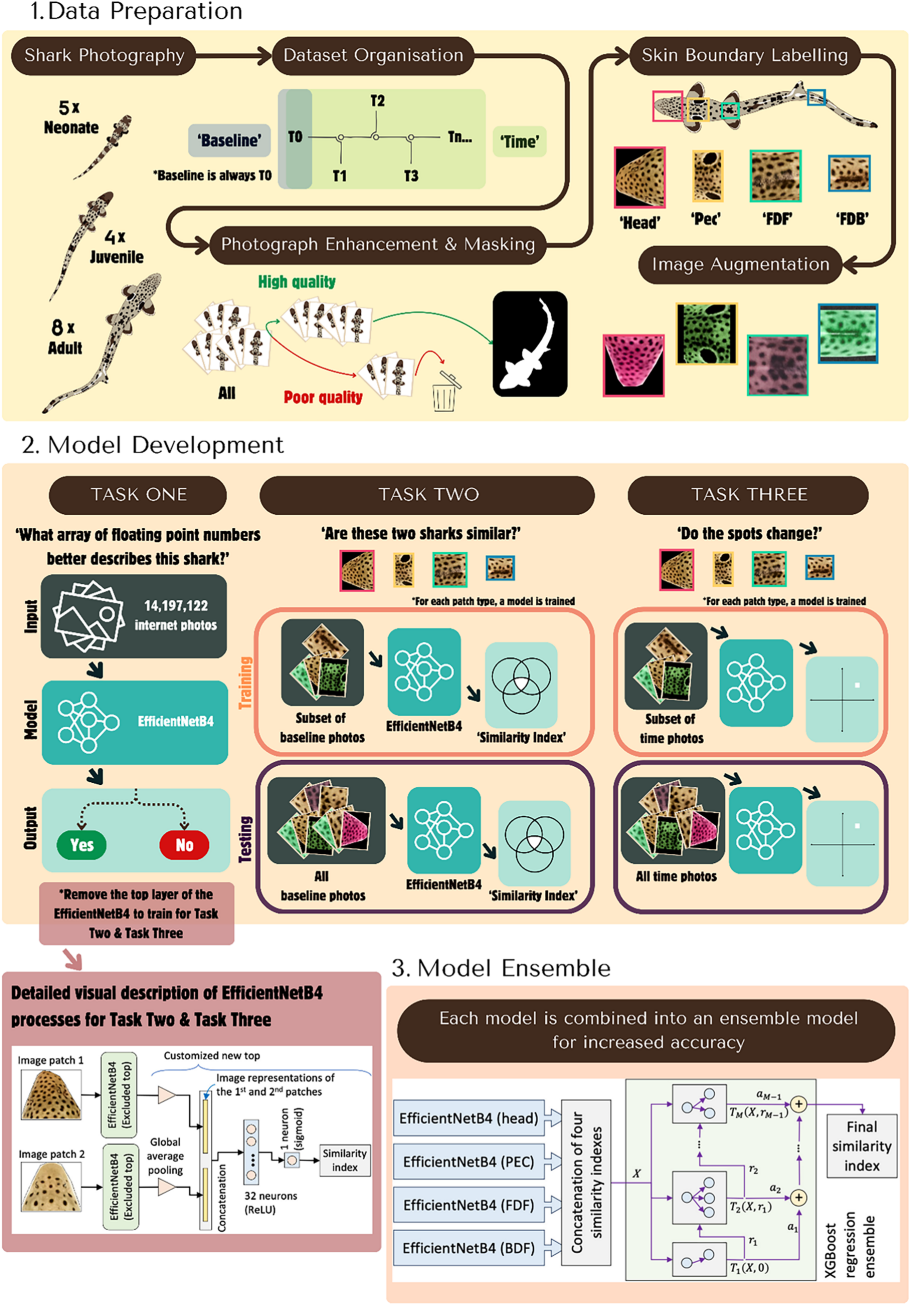
Flowchart that shows the protocol outlined in the study. From data preparation to the development and training of the models, with detailed sections on the EfficientNetB4 and *XGBoost ensemble* models.

This study presents a practical example to test photo identification for a species that has not been previously studied using this approach. This includes identifying features for ID, photographing sharks, labelling and editing photographs, and setting up a semi‐automated process to organize photographs by ID with high accuracy. Although current photo ID projects use similar methods (Gómez‐Vargas et al., 2023; Pierce et al., 2019; Schneider et al., 2022), only a few studies outline the step‐by‐step process of testing the method, compiling a database, and automating the ID process (Schneider et al., 2022). Additionally, only a few studies offer solutions that can be easily customized for different species and hypothesis (Gómez‐Vargas et al., 2023). Here we present a flowchart (Figure 9) to visualize and exemplify the protocol, from photographs, to model development, to final similarity index classifying animals by ID.

### 4.2 Data preparation: Collecting, editing, and organizing photographs

1. To test if the species has unique identification features, photographs need to be collected first for a few individuals (Pierce et al., 2019). If available, photographs of different life stages should be collected to test reliability of patterns across ontogeny. These photographs can be classified in the baseline folder.
2. Existing photographs collected through time can be classified in the time folder. This is used to test the stability of features through time (Marshall & Pierce, 2012; McCoy et al., 2018; McKinney et al., 2017). In this study, photographs of the captive animals were collected as standard research protocol. For wild populations the collection of photographs through time requires high residency and/or known aggregation sites.
3. Photographs are prepared as described in the Methods section and visualized in the flowchart (Figure 9). Importantly, the skin boundary labelling step not only allows for easier and faster model development, but it can identify which sections of the body are most indicative for ID and which sections may be most affected by changes through time.

### 4.3 Model development: Tasks 1, 2, and 3

1. Due to the highly time‐consuming task of sorting photographs, it is beneficial to develop an automated or semi‐automated model for individual recognition, even with a small dataset of photographs and only a few individuals. For instance, once the backbone of the model is in place, improvements and new individuals can always be added, and the model can be easily re‐trained when needed. This can save time, allow for re‐allocation of resources, and increase productivity for researchers.
2. Task 1 presented in the flowchart (Figure 9) shows the transfer learning approach, a fundamental step used to pre‐train the model when only a small dataset of photographs is available. Task 2 represents the standard animal identification task, which is performed by using the similarity network approach and the *XGBoost ensamble* model (see Methods sections for details on model development). Finally, the same approach is used for task 3: “Do the spots change?”

As each species is unique, the protocol suggested in this study can be customized and further improved in its applications. The controlled environment of this study represents both a strength and a limitation. The known ID of individuals, the existing record of labeled photographs, and the quality of the photographs were important factors to be able to develop the model. On the other hand, although the model was trained to work with a small dataset and with different options of photograph quality, the real‐life success of this method needs to be tested by applying the protocol to a wild population of epaulette sharks.

### 4.4 Advancements in AI applications for photo ID

The application of AI through customized deep learning models is becoming increasingly important to process and analyze long‐term datasets (Meekan et al., 2020; Schneider et al., 2022; Winton et al., 2023). Establishing a semi‐automated classifier early into the stages of a project is most beneficial in saving time when later the large volume of photographs makes visual identification more challenging. However, new photo ID projects face certain challenges in developing AI models to ID individuals from limited photographs. First, new projects, especially when working with wild populations, may have a limited number of photographs of the same animal for training the AI on ID features (Christin et al., 2019; Miele et al., 2021). Second, projects might not have access to powerful image classifiers or HPC technology, limiting the applications of AI technology. Finally, when new individuals are added to the population, models need to be fully re‐trained (Schneider et al., 2022). The accuracy of the DNN model developed in this study was a stepwise process largely attributed to the efficacy of the similarity network method and *XGBoost ensemble* model, resulting in more than 85% accuracy and 100% precision when identifying individuals from a small database.

### 4.5 The *XGBoost ensemble* model: training AI models with standard computational capabilities

The *XGBoost* approach used in this study is particularly advantageous for new photo ID projects that may lack access to HPC, as it allows for separate training of each model on standard computers. As a result, high‐precision AI models can be accessible to smaller photo ID projects and feasible for smaller Non‐Governmental organisations and community‐lead initiatives, which often have limited resources (Chin & Pecl, 2018). The implementation of the *XGBoost ensemble* model, which integrates outputs from four independently trained models, each focusing on different cropped sections of the shark's body, significantly optimized the ability of the DNN to distinguish adult individuals with high accuracy. The *XGBoost* model can assess model performance on each body section and assemble the independent models by ranking their respective performances. For instance, models with better performance are indicative of which body sections are most distinct across individuals, a valuable insight for cases where photographs capture only parts of an animal's body (Andreotti et al., 2018; Armstrong et al., 2019; Pierce et al., 2019).

### 4.6 The similarity network approach: detecting changes through time and adding new individuals

The DNN model was trained to distinguish individual epaulette sharks through a similarity network approach, assessing how similar two individuals are, and using this metric to match photographs with the correct ID (Schneider et al., 2022). Through this method, the model can be presented with new individuals, evaluating them as different from any shark it has previously learned, thereby identifying them as new sharks. Additionally, the comparison of similarity scores allows for the evaluation of morphological changes in a target species by comparing images from different time periods and analyzing the resulting similarity scores. The similarity network approach is a crucial technological advancement for addressing the key requirement for long‐term photograph identification: patterns and morphology must remain stable over time for consistent re‐identification of individuals (Ferreira et al., 2020; Marshall & Pierce, 2012).

### 4.7 Using AI to identify morphology changes through time

The best‐performing version of the DNN model confirmed that adult epaulette sharks maintained their patterns throughout the study, as the model performance remained high when identifying individuals from consecutive photographs in time. Changes in patterns through time would have appeared as a substantial change in performance score for this task. Although the efficiency of this process is yet to be validated with wild populations, it is important to consider the effect that temporal changes in morphology could have in the training of an AI model. If changes in the ID patterns do occur, the ease of re‐identification through time might be affected (Pierce et al., 2019). Additionally, if morphology does change in time, and training datasets are assembled with temporally consecutive photographs, the training of the model might be affected, and the model might not be able to accurately learn the similarities between individuals. For this reason, the similarity network approach proposed in this study can be applied by comparing photographs of the same individual through time and testing the performance of the model as a proxy of morphological stability. Overall, training on multiple photographs taken at the same time is a better approach compared to training on temporally consecutive photographs. When possible, photographs taken at the same time offer multiple examples of different lighting and angles from which models can reliably learn (Christin et al., 2019). While this is the best approach to learning features, there are other methods, such as one‐shot learning, to train a model with only one photograph (Schneider et al., 2022). For adult individuals, patterns need to be distinguishable and persistent through time (Marshall & Pierce, 2012), and these assumptions need to be tested for juveniles and immature individuals, which might have significantly different morphologies compared to adults (Bellodi et al., 2023; Fu et al., 2016).

### 4.8 Photo ID for subadults and immature life stages

When the identification protocol used on adult epaulette sharks was applied to neonates and juveniles, ID was much more challenging. Visually distinguishing epaulette sharks at early life stages was difficult, and therefore poor model performance across early ontogeny was expected (Christin et al., 2019). Consequently, photographs of juveniles and neonates had to be excluded from the training dataset. The exclusion of immature life stages from the training dataset raises the question of whether photo ID is suitable for other species that experience significant ontogenetic morphological changes during development (Ferreira et al., 2020; Marshall & Pierce, 2012). For instance, the Indo‐Pacific leopard shark (*Stegostoma tigrinum*), also called zebra sharks due to their skin patterns during the juvenile stage (Dahl et al., 2019), may be a good candidate to test the reliability of patterns for individual juveniles. As photo ID can only be performed with reliable patterns, and species might experience significant changes from their juvenile to the adult stage, it is important to consider removing those stages to prevent poor model performance. On the other hand, the protocol outlines in this study could be adapted to systematically test the reliability of patterns over ontogeny. When frequent photographs are available for each juvenile shark, AI could be trained to detect small changes through time, until changes become less and less revenant and patterns stabilize in the adult form.

## 5 CONCLUSION

Initially, the assessment of baseline conditions necessary for photo ID was conducted, followed by the development of an AI model to semi‐automate the photograph sorting process. The resulting deep learning model represents a seamless integration of established methodologies and innovative solutions, effectively identifying adult sharks and assessing the stability of their patterns over time. This study introduces a novel approach to developing a deep learning model by successfully navigating the challenges posed by small training image datasets and the absence of HPC capabilities. The solutions outlined in this study are replicable, offering a framework that can be applied to test the temporal stability of identification features for other species. This is relevant to studies currently using photo ID or exploring its applicability to new species. The next step in applying this method involves testing it with different species, varying database, and wild populations, ultimately proving its usefulness across a broader range of elasmobranch species. Tests should include both projects with long‐term, large databases and projects focusing on species that change morphology over time.

## AUTHOR CONTRIBUTIONS

M.L., J.L.R., and A.C. conceived and designed the project. Data were collected by M.L. and D.A. prepared the data for analysis. M.J. designed the AI‐powered software and analyzed the data. Figures and tables were created by M.J. and edited by S.B. The original manuscript was written by M.L. and M.J. Review and editing was performed by A.C., A.B., S.B., and J.L.R. All authors approved the final text.

## Supporting information


**TABLE S1** The capturing dates of the pictures taken from each shark in our baseline dataset (B0) and time dataset (T0, T1, T2, etc).
