# Supplementary material for: Novel use of deep neural networks on photographic identification of epaulette sharks (Hemiscyllium ocellatum) across life stages
Source: J Fish Biol. 2024 Aug 10;105(6):1572–87. doi: 10.1111/jfb.15887 (PMC11650928; doi:10.1111/jfb.15887)
Supplement: Supplementary file 1 — TABLE S1 The capturing dates of the pictures taken from each shark in our baseline dataset (B0) and time dataset (T0, T1, T2, etc). [file JFB-105-1572-s001.docx]

# Supporting Information

Table S1. The capturing dates of the pictures taken from each shark in our Baseline dataset (B0) and Time dataset (T0, T1, T2, etc.).

| **Unique shark names** | **B0** | **T0** | **T1** | **T2** | **T3** | **T4** | **T5** | **T6** | **T7** | **78** | **79** | **T10** | **T11** | **T12** | **T13** |
| --- | --- | --- | --- | --- | --- | --- | --- | --- | --- | --- | --- | --- | --- | --- | --- |
| **HO_100** | 22/12/2021 | 27/02/2020 | 1/04/2021 | 4/11/2021 | 14/12/2021 |  |  |  |  |  |  |  |  |  |  |
| **HO_102** | 22/12/2021 | 11/03/2020 | 1/04/2021 | 4/11/2021 | 14/12/2021 |  |  |  |  |  |  |  |  |  |  |
| **HO_103** | 22/12/2021 | 11/03/2020 | 1/04/2021 | 4/11/2021 | 14/12/2021 |  |  |  |  |  |  |  |  |  |  |
| **HO_105** | 22/12/2021 | 11/03/2020 | 1/04/2021 | 4/11/2021 | 14/12/2021 |  |  |  |  |  |  |  |  |  |  |
| **HO_106** | 22/12/2021 | 21/09/2020 | 20/03/2021 | 4/11/2021 | 14/12/2021 |  |  |  |  |  |  |  |  |  |  |
| **HO_107** | 22/12/2021 | 21/09/2020 | 20/03/2021 | 4/11/2021 | 14/12/2021 |  |  |  |  |  |  |  |  |  |  |
| **HO_109** | 22/12/2021 | 21/09/2020 | 20/03/2021 | 4/11/2021 | 14/12/2021 |  |  |  |  |  |  |  |  |  |  |
| **HO_110** | 22/12/2021 | 21/09/2020 | 20/03/2021 | 4/11/2021 | 14/12/2021 |  |  |  |  |  |  |  |  |  |  |
| **HO_111** | 22/12/2021 | 22/06/2021 | 19/08/2021 | 13/10/2021 | 4/11/2021 | 13/12/2021 |  |  |  |  |  |  |  |  |  |
| **HO_112** | 22/12/2021 | 22/06/2021 | 19/08/2021 | 13/10/2021 | 4/11/2021 | 13/12/2021 |  |  |  |  |  |  |  |  |  |
| **HO_113** | 22/12/2021 | 22/06/2021 | 19/08/2021 | 13/10/2021 | 4/11/2021 | 13/12/2021 |  |  |  |  |  |  |  |  |  |
| **HO_114** | 22/12/2021 | 22/06/2021 | 19/08/2021 | 13/10/2021 | 4/11/2021 | 13/12/2021 |  |  |  |  |  |  |  |  |  |
| **HO_115** | 22/12/2021 | 22/06/2021 | 19/08/2021 | 13/10/2021 | 4/11/2021 | 13/12/2021 |  |  |  |  |  |  |  |  |  |
| **HO_116** | 24/03/2023 | 22/08/2022 | 5/09/2022 | 12/09/2022 | 19/09/2022 | 10/10/2022 | 17/10/2022 | 5/12/2022 | 23/01/2023 | 6/02/2023 | 15/02/2023 | 20/02/2023 | 27/02/2023 |  |  |
| **HO_117** | 24/03/2023 | 12/08/2022 | 22/08/2022 | 29/08/2022 | 12/09/2022 | 3/10/2022 | 19/09/2022 | 10/10/2022 | 17/10/2022 | 5/12/2022 | 23/01/2023 | 6/02/2023 | 20/02/2023 | 27/02/2023 | 22/03/2023 |
| **HO_118** | 24/03/2023 | 12/08/2022 | 29/08/2022 | 5/09/2022 | 3/10/2022 | 17/10/2022 | 5/12/2022 | 6/02/2023 | 15/02/2023 | 20/02/2023 | 27/02/2023 | 22/03/2023 |  |  |  |
| **HO_119** | 24/03/2023 | 12/08/2022 | 5/09/2022 | 12/09/2022 | 3/10/2022 | 10/10/2022 | 17/10/2022 | 5/12/2022 | 23/01/2023 | 6/02/2023 | 15/02/2023 | 20/02/2023 | 27/02/2023 |  |  |
